# Negative Impact of the COVID-19 Pandemic on Admissions for Intracranial Hemorrhage

**DOI:** 10.3389/fneur.2020.584522

**Published:** 2020-09-18

**Authors:** Amr Abdulazim, Anne Ebert, Nima Etminan, Kristina Szabo, Angelika Alonso

**Affiliations:** ^1^Department of Neurosurgery, Medical Faculty Mannheim, Mannheim Center for Translational Neurosciences, Heidelberg University, Heidelberg, Germany; ^2^Department of Neurology, Medical Faculty Mannheim, Mannheim Center for Translational Neurosciences, Heidelberg University, Heidelberg, Germany

**Keywords:** COVID-19, pandemic, intracranial hemorrhage, intracerebral hemorrhage, traumatic brain bleeding

## Abstract

**Background:** The COVID-19 pandemic has seriously impacted healthcare systems worldwide. Admissions for various non-COVID-19 emergencies have significantly decreased. We sought to determine the impact of COVID-19 on admissions for intracranial hemorrhage to a German University Hospital emergency department.

**Methods:** In a retrospective analysis of admissions to the emergency department of the University Hospital Mannheim from January to June 2020 and the corresponding time period in 2019, all patients admitted for either traumatic or non-traumatic intracranial hemorrhage were evaluated. Poisson regression was performed to analyze changes in admission rates as a function of year, epoch (COVID-19-epoch, March to April 2020 and corresponding months 2019; non-COVID-19-epoch, January to February and May to June 2019/2020) and the interaction of year and epoch (reflecting the impact of the pandemic and subsequent lockdown measures).

**Results:** Overall, 320 patients were included in the study. During the COVID-19-epoch, admission rates for spontaneous intracranial hemorrhage decreased significantly by 42.1% (RR 0.579, *p* = 0.002, 95% confidence interval 0.410–0.818). Likewise, admission rates for traumatic intracranial hemorrhage decreased significantly by 53.7% [RR = 0.463, *p* < 0.001, 95% confidence interval (CI) 0.358–0.599].

**Conclusion:** The decrease of spontaneous intracranial hemorrhages may be a consequence of underutilization of the healthcare system whereas decreasing rates of traumatic intracranial hemorrhage admissions may predominantly reflect a decrease in true incidence rates due to lockdown measures with restricted mobility. Raising patient awareness to seek emergency healthcare for acute neurological deficits during lockdown measures is important to ensure appropriate emergency care for patients with intracranial hemorrhage.

## Introduction

The COVID-19 pandemic has a deep impact on healthcare systems worldwide. Analysis of emergency calls and admissions to emergency departments have recorded a pronounced increment (1, 2). In countries heavily stricken by COVID-19, rapidly rising patient numbers continue to exceed healthcare resources (2, 3) with dramatic consequences (4). At the same time, reports on decreasing admissions for non-COVID-19 conditions are becoming more frequent. While especially cerebrovascular and cardiovascular emergencies seem to be affected (5–7), reductions of trauma admissions have also been reported (8).

Intracranial hemorrhage is a heterogeneous condition, comprising both traumatic and non-traumatic disease patterns. Intracerebral hemorrhage, the leading syndrome in non-traumatic hemorrhage, is characterized by a high case fatality and a high disease burden in survivors, mostly causing a life in functional dependence (9). Whereas the risk of both spontaneous and traumatic intracranial hemorrhage increases with age, traumatic brain injury with intracranial bleeding complications also affects the younger (10) with substantial loss of disability-adjusted life-years. In both cases, an optimal emergency care is of utmost importance to attenuate a potentially fatal course of disease.

We sought to determine the impact of the COVID-19 pandemic and subsequent lockdown measures on admission rates for intracranial hemorrhage during the weeks of the initial spreading of COVID-19 and after stabilization of infection rates in a University Hospital in Germany.

## Methods

The local ethics committee (Ethikkommission II der Medizinischen Fakultät Mannheim, Universität Heidelberg) approved this retrospective study. Patient consent was waived due to the retrospective character of the study and the lack of patient interaction.

The study comprises two observation periods, January to June 2020 and the corresponding period in 2019. The months March and April 2020 were defined as the lockdown period: daily infection rates with SARS-CoV-2 were recorded by the Robert Koch Institute from March 2nd on, and partial lockdown measures were implemented on March 9th. Emergency and pandemic plans in the University Hospital Mannheim were updated on February 28th, and a partial ban of visitors was realized on March 5th.

March and April 2019 and 2020 were termed epoch 1, the remaining months of the years 2019 and 2020 were termed epoch 2. All patients admitted to the emergency department with a diagnosis of intracranial hemorrhage were identified by a retrospective chart review of the admissions to our interdisciplinary emergency department. In detail, all emergency department charts with an ICD-10 admission diagnosis of I60ff, I61ff, I62ff, and S06ff were screened. Inclusion criterion was admission for spontaneous or traumatic intracranial hemorrhage, comprising intracerebral, subdural, epidural, intraventricular and/or subarachnoid hemorrhage. Traumatic hemorrhages affecting more than one intracranial compartment were categorized as complex traumatic brain injury. Patients with intracranial hemorrhage due to arteriovenous malformation or fistula, cerebral venous thrombosis, underlying cavernoma or intracranial aneurysms and patients with hemorrhagic intracerebral malignoma were excluded. The rationale for exclusion of these patient groups is the significant heterogeneity in terms of epidemiology, risk factors, treatment options and prognosis together with low absolute numbers, thus precluding a statistically appropriate evaluation.

### Statistical Analysis

Statistical analysis was performed using IBM SPSS Statistics Version 25. Poisson regression was used to test if the rate of admissions changed as a function of year, epoch (epoch 1, March–April; epoch 2, January–February + May–June) and year-by-epoch interaction (reflecting the impact of the pandemic and subsequent lockdown measures), expressed as rate ratio (RR) along with its 95% confidence interval. A *p*-value < 0.05 was considered as statistically significant. For group comparisons between the lockdown period (March-April 2020) and the corresponding period in 2019 Chi-square test was used.

## Results

Over the cumulative observation periods 01-06/2019 and 01-06/2020, 320 patients were admitted with the diagnosis of either spontaneous or traumatic intracranial hemorrhage (mean age 69.4 years, 59.7% male; see Table 1) with a mean monthly admission rate of 26.67 cases [standard deviation (SD) ± 6.050]. Non-traumatic intracranial hemorrhages, predominantly spontaneous intracerebral hemorrhages, comprised 43.1% of the cases, and 40.6% of patients were on some form of anticoagulant medication at the time of admission (Table 1).

**Table 1:** Characteristics of patients admitted for intracranial hemorrhage between January and June in 2019 and 2020, as well as in March/April in 2019 and 2020.

	**Total Jan–June 2019 + Jan–June 2020**	**March/April 2019**	**Lockdown 2020**
	***n* = 320**	***n* = 54**	***n* = 33**
Sex (male, *n*; %)	191 (59.7)	33 (61.1)	21 (63.6)
Age (mean, range)	69.4 (18–101)	69.2 (30–96)	68.4 (18–101)
Non-traumatic intracranial hemorrhage (*n*, %)	138 (43.1)	19 (35.2)	11 (33.3)
ICH	129	18	9
IVH	3	1	1
SAH	6	0	1
Traumatic intracranial hemorrhage (*n*, %)	182 (56.9)	35 (64.8)	22 (66.7)
cTBI	35	4	8
tICH	36	8	4
tSDH	74	13	8
tSAH	31	7	2
EDH	6	3	0
Anticoagulation (*n*, %)	130 (40.6)	19 (35.2)	14 (42.4)
NOAC	40	5	5
Phenprocoumon	16	3	2
PAI	64	9	7
Dual PAI	5	0	0
Other^*^	5	2	0

* *Enoxaparin, 3; Nadroparin, 1; Fondaparinux, 1*.

*ICH, intracerebral hemorrhage; IVH, intraventricular hemorrhage; SDH, subdural hematoma; SAH, non-aneurysmatic subarachnoid hemorrhage; cTBI, complex traumatic brain injury; tICH, traumatic intracerebral hemorrhage; tSDH, traumatic subdural hematoma; tSAH, traumatic subarachnoid hemorrhage; EDH, epidural hematoma; NOAC, non-vitamin K antagonist oral anticoagulants; PAI, platelet aggregation inhibitors*.

**Figure 1:**
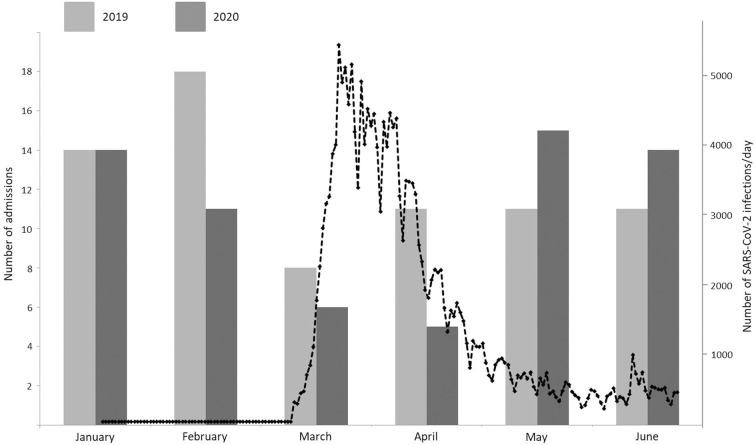
Monthly admission rates for spontaneous intracranial hemorrhage between January and June 2019 (light gray) and January and June 2020 (dark gray) and daily new infections in Germany with SARS-CoV-2 from March 2nd to June 30th (black dotted line).

**Figure 2:**
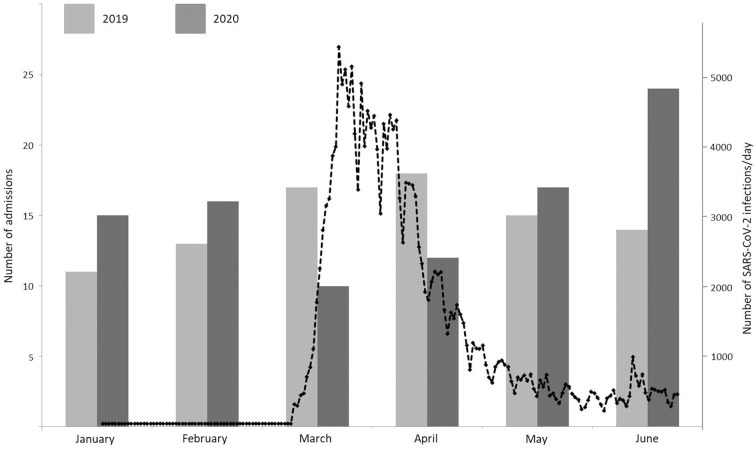
Monthly admission rates for traumatic intracranial hemorrhage between January and June 2019 (light gray) and January and June 2020 (dark gray) and daily new infections in Germany with SARS-CoV-2 from March 2nd to June 30th (black dotted line).

During the lockdown period (03-04/2020), admission rates for spontaneous intracranial hemorrhage decreased significantly by 42.1%, as found in a significant year-by-epoch interaction [RR = 0.579, *p* = 0.002, 95% confidence interval (CI) 0.410–0.818; see Figure 1]. Likewise, admission rates for traumatic intracranial hemorrhage decreased significantly by 53.7%, as found in a significant year-by epoch interaction [RR = 0.463, *p* < 0.001, 95% confidence interval (CI) 0.358–0.599; see Figure 2].

The treatment rate with any anticoagulant did not vary between the two observation periods, neither for spontaneous (*p* = 0.91) nor for traumatic intracranial hemorrhage (*p* = 0.42).

In 2020 admission rates increased after the lockdown period for both spontaneous and traumatic intracranial hemorrhage. For traumatic hemorrhages, there was a significant effect for “year” in the Poisson regression (RR = 1.358, *p* = 0.007, CI 1.089–1.695). As reflected in Figure 2, this effect is mainly driven by the excess admission rates in June.

## Discussion

Our data illustrate the significant impact of the COVID-19 pandemic and associated lockdown measures on admission rates for non-traumatic and traumatic intracranial hemorrhage, with a 42.1% decrease and 53.7% decrease, respectively, in March and April 2020. The referenced period is characterized by the peak of SARS-CoV-2 infection rates in Germany, exceeding 1,000 infections per day on March 8th for the first time, rising to a peak of nearly 5,500/day on March 16th, and stable infection rates of <1,000/day since May 1st. The year-by-epoch interaction resulting from Poisson regression is a good index of a causal relationship with the COVID-19 pandemic.

Two factors may have caused the pronounced decrease observed: a decrease in true incidence rates of intracranial hemorrhages, or a decrease in utilization of the health care system. For spontaneous intracranial hemorrhage, a COVID-19-related decrease in incidence rates is highly unlikely. However, similarly, a significant decrease in admission rates has also been reported for other cerebro- and cardiovascular emergencies, such as ischemic stroke (5, 6, 11), transient ischemic attack (12) or acute coronary syndrome (7) in several countries. Various patient-centered factors may contribute to this phenomenon. Insistent stay-at-home campaigns and public discussions about imminent intensive care bottlenecks may have unsettled patients whether to present to hospital. In addition, a reluctance to seek medical, especially hospital care, may result from a fear of infection, together with concerns of being isolated due to a strict ban of visitors since the midst of March.

In case of traumatic intracranial hemorrhages, the lockdown status may well have contributed to a decrease in true incidence rates. The observation period March–April 2020 coincides with nation-wide partial lockdown measures in Germany, including social distancing, self-isolation, quarantining, but also a travel ban, closing of schools/daycare and public facilities, such as fitness centers. These measures correspond with a significant decrease in mobility as reflected in freely available online mobility data^1^ As a consequence, admissions for extracranial trauma have equally dropped in a significant manner (8, 13). Of interest, our study identified a significant increase in admissions for traumatic intracranial hemorrhage in 2020 compared to the preceding year when excluding the COVID-periods March–April 2020 and the corresponding months in 2019. The increase is mainly driven by excess admission rates during June 2020, possibly attributable to a catch-up effect after a relaxation of lockdown measures.

Overall, the significant increase in admissions for traumatic intracranial hemorrhage in the post-lockdown months May and June 2020 suggests rather a shift of admissions than missed admissions during the lockdown period. However, the decrease of admissions in non-traumatic intracerebral hemorrhage needs to be regarded with concern. Timely diagnosis and treatment of intracranial hemorrhage is indispensable to keep morbidity and mortality low. Although actual mortality rates in Germany do not show an excess mortality since the beginning of the COVID-19 pandemic, the sequelae of missed admissions for cerebro- and cardiovascular emergencies may still lie ahead. A *de novo* absence of patients with intracranial hemorrhages in case of a second wave of infections has to be prevented by all means. This issue has been addressed by the German Society for Neurology demanding campaigns to inform the public about the priority of stroke care in times of the pandemic in order to avoid serious healthcare consequences^2^.

## Data Availability Statement

The raw data supporting the conclusions of this article will be made available by the authors, without undue reservation.

## Ethics Statement

The studies involving human participants were reviewed and approved by Ethikkommission II der Medizinischen Fakultät Mannheim, Universität Heidelberg. Written informed consent for participation was not required for this study in accordance with the national legislation and the institutional requirements.

## Author Contributions

AAb: data analysis and interpretation, drafting manuscript, and final approval. AE: statistical analysis, critical revision of manuscript, and final approval. NE and KS: data analysis and interpretation, critical revision of manuscript, and final approval. AAl: concept and design, data acquisition, data analysis and interpretation, drafting manuscript, and final approval. All authors contributed to the article and approved the submitted version.

## Conflict of Interest

The authors declare that the research was conducted in the absence of any commercial or financial relationships that could be construed as a potential conflict of interest.

## Footnotes

^1^https://www.covid-19-mobility.org/

^2^https://www.dgn.org/rubrik-dgn/stellungnahmen-der-dgn/3919-auswirkungen-der-sars-cov-2-pandemie-auf-die-versorgung-zerebrovaskulaerer-erkrankungen
